# Extracellular vesicles derived from endothelial cells modulate macrophage phenotype in vitro

**DOI:** 10.1186/s40001-023-01427-6

**Published:** 2023-11-09

**Authors:** Zhizhen He, Johannes Greven, Yulong Shi, Kang Qin, Qun Zhao, Xing Zhang, Eva Miriam Buhl, Jörg Eschweiler, Frank Hildebrand, Elizabeth Rosado Balmayor

**Affiliations:** 1 Department of Orthopedics, Trauma and Reconstructive Surgery, RWTH Aachen University Hospital, Pauwelsstraße 30, 52074 Aachen, Germany; 2 Division of Joint Surgery and Sports Medicine, Department of Orthopedic Surgery, Zhongnan Hospital of Wuhan University, Wuhan, 430071 China; 3 Electron Microscopy Facility, Institute for Pathology, RWTH Aachen University Hospital, Pauwelsstraße 30, 52074 Aachen, Germany; 4 Experimental Orthopaedics and Trauma Surgery, RWTH Aachen University Hospital, Pauwelsstraße 30, 52074 Aachen, Germany

**Keywords:** Macrophages polarization, Extracellular vesicles, Endothelial cells, NFκB pathway

## Abstract

Extracellular vesicles (EVs) mediate cell-to-cell communication by horizontally transferring biological materials from host cells to target cells. During exposure to pathogens, pathogen-associated molecular patterns (e.g., lipopolysaccharide, LPS) get in contact with endothelial cells and stimulate the secretion of endothelial cell-derived EVs (E-EVs). The triggered EVs secretion is known to have a modulating influence on the EVs-receiving cells. Macrophages, a major component of innate immunity, are polarized upon receiving external inflammatory stimuli, in which toll-like receptor4 (TLR4)—nuclear factor kappa B (NFκB) pathway plays a key role. However, the functions of LPS-induced E-EVs (E_LPS_-EVs) in modulating macrophage phenotype and activation remain elusive. We collected the EVs from quiescent endothelial cells (E_Nor_-EVs) and E_LPS_-EVs to detect their stimulatory role on NR8383 macrophages. Isolated EVs were characterized by transmission electron microscopy (TEM), western blot assay, and nanoparticle tracking analysis (NTA). NR8383 macrophages were stimulated with ELPS-EVs, ENor-EVs, or PBS for 24 h. Hereafter, the uptake of EVs by the macrophages was investigated. Upon EVs stimulation, cellular viability was determined by MTT assay, while macrophage phenotype was analyzed by flow cytometry and immunofluorescence analysis. Furthermore, a western blot assay was conducted to evaluate the potentially involved TLR4–NFκB pathway. Interestingly, upon exposure to LPS, endothelial cells secreted significantly higher amounts of EVs (i.e., E_LPS_-EVs) when compared to quiescent cells or cells in PBS. The E_LPS_-EVs were also better internalized by NR8383 macrophages than E_Nor_-EVs. The cellular viability of E_LPS_-EVs-treated macrophages was 1.2 times higher than those in the E_Nor_-EVs and PBS groups. In addition, E_LPS_-EVs modulated NR8383 macrophages towards a proinflammatory macrophage M1-like phenotype. This was indicated by the significantly upregulated expressions of proinflammatory macrophage biomarkers CD86 and inducible nitric oxide synthase (iNOS) observed in E_LPS_-EVs-treated macrophages. The TLR4–NFκB signaling pathway was substantially activated in E_LPS_-EVs-treated macrophages, indicated by the elevated expressions of makers TLR4 and phosphorylated form of nuclear factor kappa B p65 subunit (p-NFκBp65). Overall, our results indicate that E-EVs play a crucial role in macrophage phenotype modulation under inflammatory conditions.

**Supplementary Information:**

The online version contains supplementary material available at 10.1186/s40001-023-01427-6.

## Introduction

Following vascular injury and infection, vascular endothelial cells are known to attract circulating monocytes [1]. Once the monocytes penetrate the vascular wall, differentiation into macrophages occurs, which is primarily responsible for triggering the inflammatory reaction to counteract pathogens, restore damaged tissue, and/or enhance the immunocompetence of other cell types [2, 3]. Being exposed to bacterial lipopolysaccharide (LPS), macrophages present a proinflammatory phenotypic status (M1) as a part of the immune response, secreting proinflammatory cytokines and shedding EVs for immune defense and cell–cell interactions.

EVs are mainly involved in intercellular communication and modulate various cellular responses. Originating from virtually any cell type, EVs are highly heterogeneous vesicles with sizes up to 10,000 nm [4]. They are commonly classified into apoptotic bodies, microvesicles, and exosomes [5]. Secreted EVs can be transported into the recipient cells, thereby modulating selected targets and cellular pathophysiological processes by the so-called horizontal transfer of their cargo [6]. Most fully developed tissues have endothelial cells (ECs) in a quiescent state, essential for their role as a barrier and signaling interface. Necessary signals are continually initiated and received by the quiescent ECs, which can be activated as needed [7]. Furthermore, endocrine, autocrine, paracrine, mechanical, and endothelium metabolic signals all play a role in keeping ECs in a quiescent state [8, 9]. Although little amounts of EVs are shed in the quiescent state, they nonetheless have significant regulatory potential for other immune cells [3]. EVs shed by quiescent ECs, may mirror a steady state of the cell creating a non-influenced cargo dependent on external triggers.

Endothelial cell-derived EVs (E-EVs) are increasingly released due to endothelial activation during inflammation and are known to affect hemostasis, different aspects of inflammatory reactions, vessel formation, apoptosis, and cell survival, as well as the differentiation and function of endothelial cells [10]. E-EVs have been of rising interest because they are shed directly into the bloodstream and can interact with various circulating cell types. They are known to also travel systemically to distant sites. It has been shown that EVs and their cargo can be influenced by present homeostatic changes, e.g., inflammatory processes [11–16]. For example, E-EVs secreted upon necrosis factor-α (TNF-α) stimulus promote inflammation and induce the release of ICAM-1 and procoagulants [11–13]. They also contribute to endothelial cell dysfunction and proinflammatory cytokine release [14, 15], inducing plasmacytoid dendritic cell maturation and the expression of inflammatory cytokines [16]. In addition, LPS-induced endothelial cells exosomes are known to promote proliferation and apoptosis resistance in artery smooth muscle cells [17], induce lung endothelium barrier disruption [18], and highly prompt vascular endothelial growth factor B (VEGF-B) expression in vascular smooth muscle cells [19].

E-EVs from inflammatory backgrounds related to illness are preferentially absorbed by monocytes compared to other immune cells such as neutrophils or lymphocytes, causing trans-endothelial migration and inflammatory reaction of EVs-receiving monocytes [20]. This shows that EVs may play a crucial role in modulating macrophage phenotype. For instance, EVs secreted by endothelial cells transduced with Krüppel-like factor 2 (KLF2) protect against atherosclerosis by shifting from proinflammatory M1 to anti-inflammatory M2 macrophages [21]. While on the other hand, oxidized low-density lipoprotein-induced E-EVs drive the polarization of monocytes/macrophages from anti-inflammatory M2 macrophages towards proinflammatory M1 macrophages [21]. However, it is still unclear how LPS- stimulated E-EVs influence macrophage phenotype.

Toll-like receptors (TLRs) belong to the essential signaling mechanism among the signaling pathways that affect macrophage function [22]. Moreover, TLR signals exert a crucial role in macrophage polarization [23]. In particular, lipopolysaccharides (LPS) bind to TLR4 driving macrophage development toward the M1 phenotype and triggering signaling cascades (e.g., nuclear factor kappa B, NF-κB) that result in the release of proinflammatory cytokines [24]. In earlier studies, we found that EVs shed by LPS-induced macrophages were successfully internalized by these cells driving them to a proinflammatory phenotype via TLR4–NFκB signaling pathway [25]. In this study, we aimed to investigate the function of LPS-stimulated E-EVs in endothelium–macrophage communication. The obtained results suggest that E-EVs and their biological material cargo significantly impact macrophage phenotype. This might highlight a possible impact of EV alterations on the development of infectious diseases. Furthermore, the results reported here represent a first step in understanding the development of local to systemic immune reactions based on EV cell–cell communication.

## Materials and methods

### Rat aortic endothelial cells culture and stimulation

Rat aortic endothelial cells (RAOEC) were obtained from Cell Applications Inc. (San Diego, CA, USA). RAOEC were cultured in a culture medium for rat endothelial cells (Cell Applications Inc.) at 37 °C in 5% CO_2_. Near-confluent RAOEC were placed in an EV-free growth medium. This medium was obtained by removing the EV contaminants in rat endothelial cell growth media by means of centrifugation at 20,000 × g for 90 min. The contaminants were discarded. Next, 100 ng/ml LPS (Sigma-Aldrich, St. Louis, MO, USA) was added to the RAOEC culture and the culture was maintained for a further 24 h. Thereafter, the cell culture supernatant was collected for E_LPS_-EVs isolation. Quiescent RAOEC were used to isolate E_Nor_-EVs.

### E-EVs isolation

Collected cell culture supernatants were sequentially centrifuged following our previously established protocol [25]. EV contaminants such as cell fragments and apoptotic bodies were excluded by two subsequent centrifugation steps starting at 300 × g for 10 min and followed by 2,000 × g for 15 min. Thereafter, EVs were pelleted by centrifugation at 20,000 × g for 90 min at 4 °C. Obtained EVs were suspended in PBS and frozen at −80 °C until further use. All EVs samples used in this study were stored for less than 2 weeks before being used again.

### NR8383 macrophages stimulation

NR8383 macrophages were obtained from Cell Applications Inc. Ham's F12K medium containing 2 mM L-glutamine and 1.5 g/L sodium bicarbonate was supplemented with 15% heat-inactivated fetal bovine serum, 100 U/mL of penicillin, and 100 μg/mL of streptomycin. The supplemented medium was used to culture the NR8383 macrophages. Cell culture media and supplements were purchased from Gibco^™^ (Waltham, MA, USA). NR8383 macrophage culture was performed at 37 °C under 5% CO_2_ and 85% humidity. After culturing NR8383 macrophages to approximately 70% confluence in cell culture dishes, cells were then stimulated with obtained E_LPS_-EVs, E_Nor_-EVs, or with equal volume of PBS. The stimulation was performed for 24 h before evaluation.

### EVs uptake assay

Obtained EVs were fluorescently labeled with the aim of studying the macrophage incorporation of these particles. For this, EVs were incubated with wheat germ agglutinin (WGA, Alexa Fluor^™^ 594 conjugate, Thermo Fisher Scientific, Waltham, MA, USA) for 30 min at 37 °C. Next, a purification was performed by means of centrifugation at 20,000 × g for 120 min at 4 °C. Fluorescently tagged EVs were then cultured with NR8383 macrophages for 24 h. DAPI was used to stain the cell nuclei (Thermo Fisher Scientific) and cells were fixed using 4% paraformaldehyde. Cells were observed and imaged in a FSX-100 Olympus microscope (Tokyo, Japan).

### EVs visualization and identification

Upon different endothelial cell stimuli, obtained EVs were examined by transmission electron microscopy (TEM) and nanoparticle tracking analysis (NTA). Features such as population, size, and morphology were investigated. In short, EVs were centrifuged into pellets and then treated with a 2.5% glutaraldehyde solution (Sigma-Aldrich) at 4 °C overnight. The EVs were cultured on grids coated with glow-discharged formvar carbon (Nickel Grid 200 mesh, Electron Microscopy Sciences, Hatfield, PA, USA) for 5 min. The grids were washed three times with distilled water. Uranyl (0.5%) was applied for negative staining. Filter paper was used to remove excess liquid. The grids were dried by air for 10 min. Samples were photographed using a LEO 906 E transmission electron microscope (Zeiss, Oberkochen, Germany) and operated at an acceleration voltage of 60 kV [26].

The concentration (particles/mL) and sizes (nm) of obtained EVs were determined by the NanoSight NS 300 system (Malvern Panalytical Ltd., Malvern, United Kingdom) following the manufacturer's instructions. For this measurement, an EV suspension was prepared using isolated EVs from 1 ml culture medium that were resuspended in 1 ml PBS. Hereafter, the obtained EV concentration values served for determining the amount of EVs used in subsequent experiments.

The total protein concentration of the EVs was assessed by means of a BCA Protein Quantification Assay Kit (Thermo Fisher Scientific). Furthermore, CD31 and CD63 were used as markers to confirm endothelial origin of the obtained EVs by means of western blot analysis.

### MTT assay

The cell proliferation of NR8383 macrophages upon 24 h of incubation with the obtained EVs was evaluated using an MTT assay. The incubation was performed under routine cell culture conditions that is at 37 °C, 5% CO_2,_ and 85% humidity. For MTT, NR8383 macrophages were incubated in serum‐free Ham's F12K medium solution containing the MTT reagent (3-(4,5-dimethylthiazol-2-yl)-2,5-diphenyltetrazolium bromide, 0.5 mg/mL) for 4 h in a light-protected environment. Thereafter, the cell culture medium was discarded, and each well received 100 μl of DMSO. Gentle rotation was utilized to guarantee that the formed precipitate had completely dissolved. A BioTek Synergy multiplate reader (BioTek, Winooski, VT, USA) was used to evaluate the absorbance of MTT at 450/620 nm. The ratio of absorbance values obtained from cells with and without EVs stimulation was used to define the viability [27].

### Immunofluorescence assay of macrophage subtype

Antibodies iNOS-FITC (1:50, Novus Biologicals, USA) were used to identify NR8383 macrophages. After being treated with E_Nor_-EVs, E_LPS_-EVs, or PBS, the NR8383 macrophages were fixed in 4% paraformaldehyde for 10 min and washed three times in PBS. After permeabilization with 0.1% Triton X-100 for 15 min and blocking with 5% BSA for 1 h at room temperature, the cells were incubated with the antibody for 20 min. After rinsed with PBS, the slides were stained with DAPI medium (Invitrogen, USA) and imaged using a fluorescent microscope.

### Flow cytometry analysis of macrophage subtype

Antibodies F4/80-PE and CD86-APC (Biolegend, San Jose, CA, USA) were used to identify NR8383 macrophages. For this, cells were enzymatically detached from the culture dish, pipetted up and down, and centrifuged at 300 × g for 4 min. The obtained cell pellet was washed twice with PBS and subsequently resuspended at 2 × 10^6^ cells/mL using ice-cold eBioscience^™^ flow cytometry staining buffer (Thermo Fisher Scientific) to obtain a single-cell suspension. Next, 10% goat serum was added to the cell suspension and incubated for 15 min. Antibody mixtures were added and a further incubation step was performed on ice for 30 min. Lastly, two washing steps were performed using ice-cold eBioscience^™^ flow cytometry staining buffer (ThermoFisher). Samples were analyzed using the BD LSR II system (BD Biosciences). Flowjo software (Tree Star, San Carlos, CA, USA) was used to generate and treat the obtained data.

### Western blot analysis: EVs characterization and macrophage differentiation-associated pathway identification

Ten percent SDS-PAGE gels were used to separate equal amounts of total protein from EV samples or cell lysates. PVDF membranes (PerkinElmer, Waltham, MA, USA) were used for protein transfer. Antibodies targeting CD63 (1:2000, rabbit, Thermo Fisher Scientific), CD31 (1:2000, rabbit, Thermo Fisher Scientific), GAPDH (1:2000, rabbit, Thermo Fisher Scientific), TLR 4 (2 µg/mL, rabbit, Thermo Fisher Scientific), p-NFκB-p65 (1:1000, Rabbit, Thermo Fisher Scientific) were used to incubate with the membranes at 4 °C overnight after blocking for 2 h with blocking solution (5% skim milk) at room temperature. Afterward, three washing steps using TBS with Tween™ buffer (TBST, Thermo Fisher Scientific) were performed. Subsequently, the membranes were incubated with the secondary antibody coupled to horseradish peroxidase (1:1,000) for 1 h at room temperature. A detection system (ECL Plus, Thermo Scientific, Waltham, MA, USA) was used to visualize immuno-reactive bands. The density of regarding bands was evaluated with the ImageJ software (Image J 1.48v, NIH, USA). Protein expression is presented as target/reference density values ratio.

### Statistical analysis

Data are presented as the means ± SD from at least three independent experiments. Student's *t*-test was used for a single comparison, and one‐way ANOVA with Bonferroni's correction was applied for multiple comparisons. Statistical analyses were performed using the software GraphPad Prism version 8 (GraphPad Software, San Diego, CA, USA). *p* < 0.05 were regarded as significant.

## Results

### EVs isolation and characterization

**Fig. 1:**
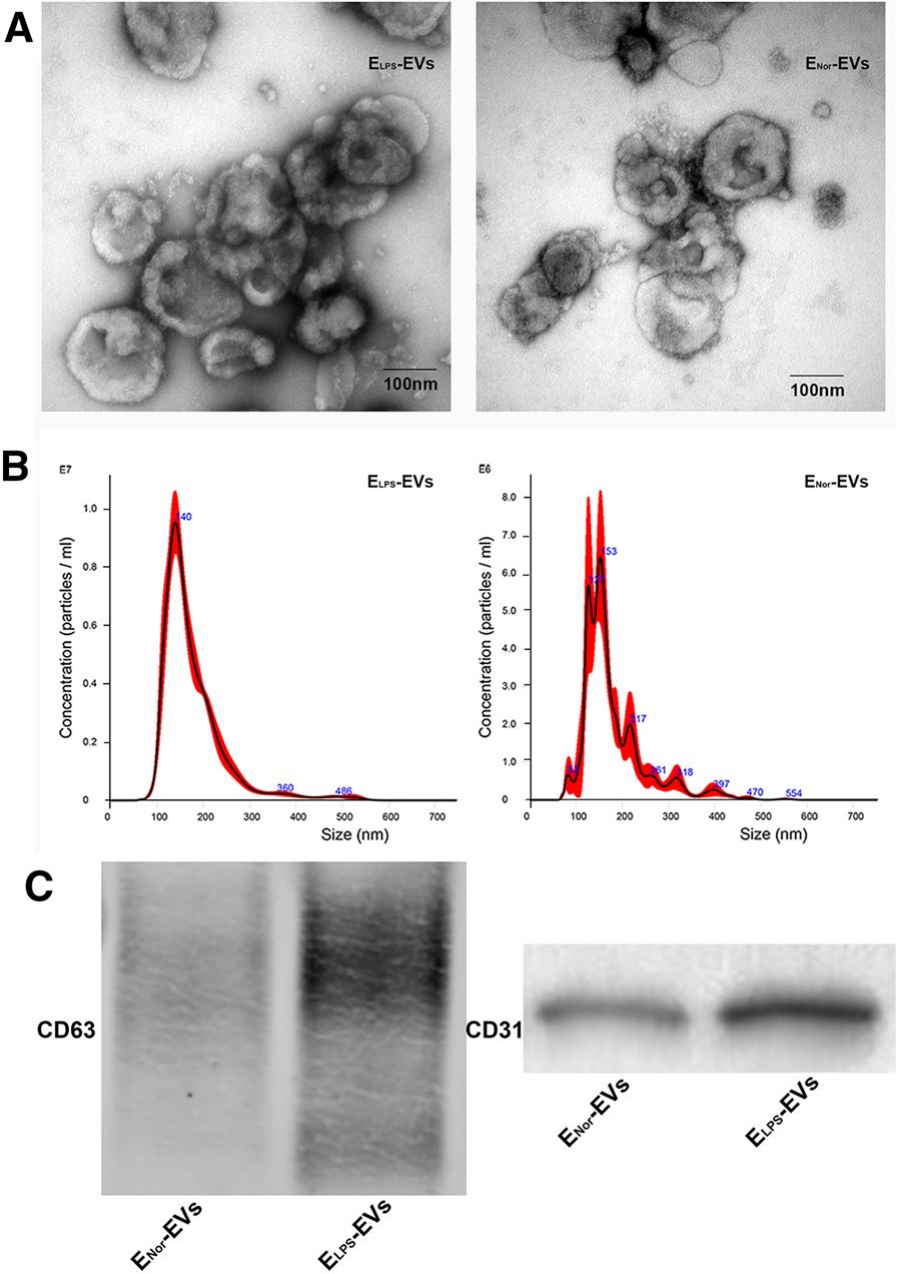
Characterization of isolated E-EVs. **A** TEM images of a representative sample of the isolated E_LPS_-EVs and E_Nor_-EVs. Micrographs revealed a circular and double-membrane structure characteristic of EVs. **B** NTA determinations of size (nm) and concentration (particles/ml) of isolated E_LPS_-EVs and E_Nor_-EVs. **C** Identification of EVs protein markers CD63 and CD31

Two different EVs samples were obtained in this study. LPS-stimulated RAOECs were used to harvest E_LPS_-EVs while quiescent RAOECs were used to isolate E_Nor_-EVs. TEM images of isolated E_LPS_-EVs and E_Nor_-EVs revealed typical EV spheroidal form with a double-membrane structure (Fig. 1A). No morphological differences could be concluded from the TEM micrographs when comparing E_LPS_-EVs and E_Nor_-EVs. NTA analysis revealed that LPS-stimulated RAOECs were able to produce 1.67-fold more EVs (i.e., E_LPS_-EVs) than quiescent RAOECs in the E_Nor_-EVs group (*p* = 0.037). The purified E_LPS_-EVs fraction had a mean size of 177 ± 3.8 nm and a concentration of 8.48 ± 0.2 (× 10^8^) particles/ml. Similar to this size range, the E_Nor_-EVs fraction featured a size of 180 ± 2.6 nm. Interestingly, the concentration of purified E_Nor_-EVs was 5.08 ± 0.6 (× 10^8^) particles/ml (Fig. 1B). This was lower than the obtained amount of particles per ml for E_LPS_-EVs. E-EVs concentration was adjusted to the needed values for each subsequent experiment accordingly.

To further characterize the obtained E-EVs in terms of purity, known EVs transmembrane protein markers CD63 (non-tissue specific) and CD31 (endothelial cell specific) were investigated in purified E_LPS_-EVs and E_Nor_-EVs samples. Figure 1C depicts positive CD63 and CD31 expression in E_LPS_-EVs and E_Nor_-EVs lysates, indicating the presence of the lipid-bilayer structure specific of EVs as well as confirming the endothelial cell origin. The original western blot images are available in the supplementary materials (Additional file 1: Figure S1).

### EVs internalization by NR8383 macrophages: effect on cell viability

**Fig. 2:**
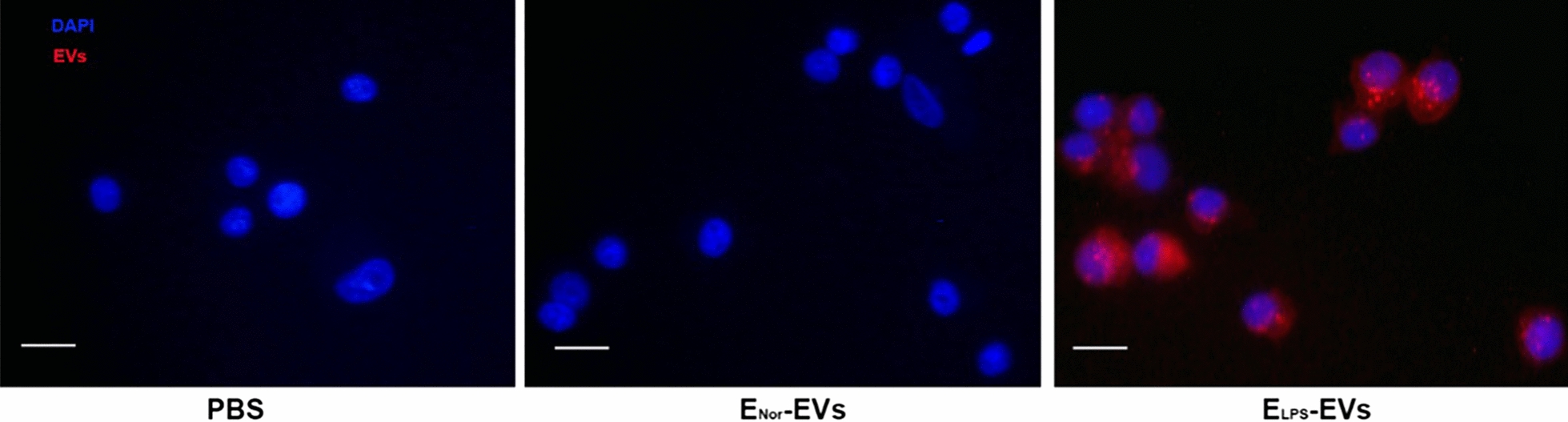
E-EVs Incorporation by NR8383 macrophages. Immunofluorescent microscopy images show that fluorescently labeled EVs were internalized by NR8383 macrophages only when E_LPS_-EVs were used. Negligible signal was observed for E_Nor_-EVs, indicating no internalization. In the images, red fluorescence corresponds to the fluorescently labeled E-EVs, while blue DAPI indicates cellular nuclei. Used magnification was × 400. Scale bar: 10 µm

**Fig. 3:**
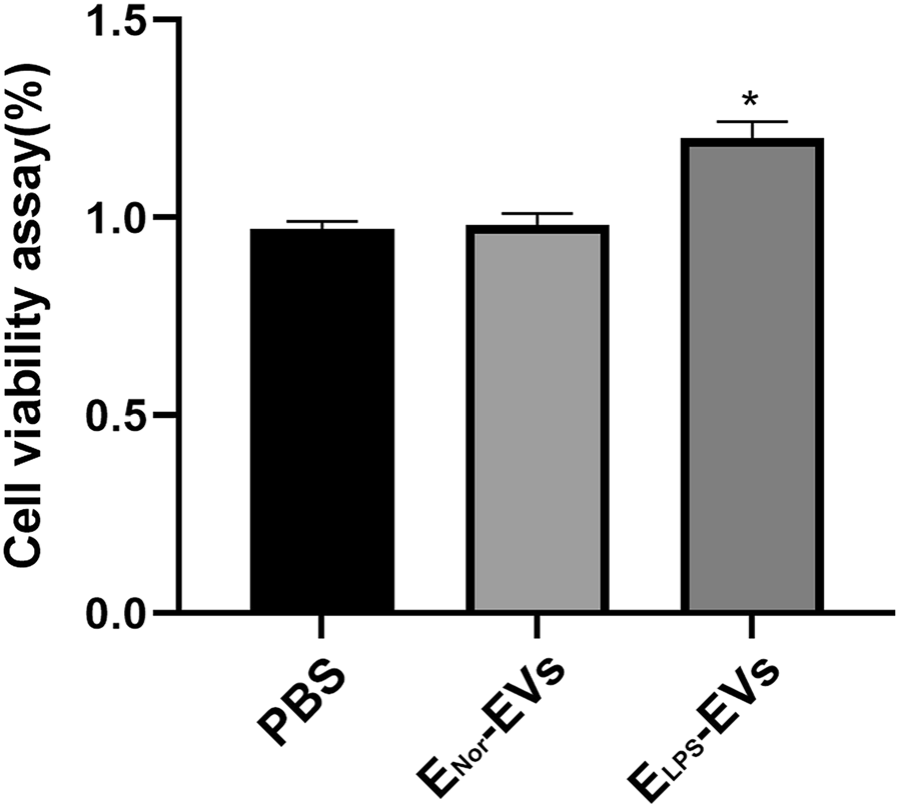
NR8383 macrophages viability upon stimulation with E-EVs. MTT assay was performed to evaluate the cell viability of NR8383 macrophages cultured for 24 h in the presence of E_LPS_-EVs or E_Nor_-EVs. n = 5 replicates were used for these experiments. *p < 0.05 E_LPS_-EVs compared to control, PBS group and E_Nor_-EVs group. No significant difference was found in cell viability when comparing the PBS and E_Nor_-EVs groups, p > 0.05. Statistical analysis was performed by one‐way ANOVA with Bonferroni's correction

The internalization of isolated E-EVs by NR8383 macrophages was assessed by immunofluorescence microscopy. Representative image in Fig. 2 revealed the successful internalization of WGA-labeled E_LPS_-EVs (red) by NR8383 macrophages by a clear red fluorescence signal located within the cells (identified by blue DAPI signal). Interestingly, failed internalization was observed for WGA-labeled E_Nor_-EVs by NR8383 macrophages, as negligible red fluorescence signal was obtained (Fig. 2). Next, the effect of E-EVs internalization on the cell viability of NR8383 macrophages was evaluated. Cell viability was not affected in any of the investigated groups (i.e., E_LPS_-EVs, E_Nor_-EVs, or with plain PBS.) Remarkably, there was a significant increase in cell viability of the NR8383 macrophages after stimulation with E_LPS_-EVs when compared to the cells in the E_Nor_-EVs and PBS groups (*p* < 0.0001, Fig. 3).

### Modulation of NR8383 macrophage phenotype upon EVs stimulation

**Fig. 4:**
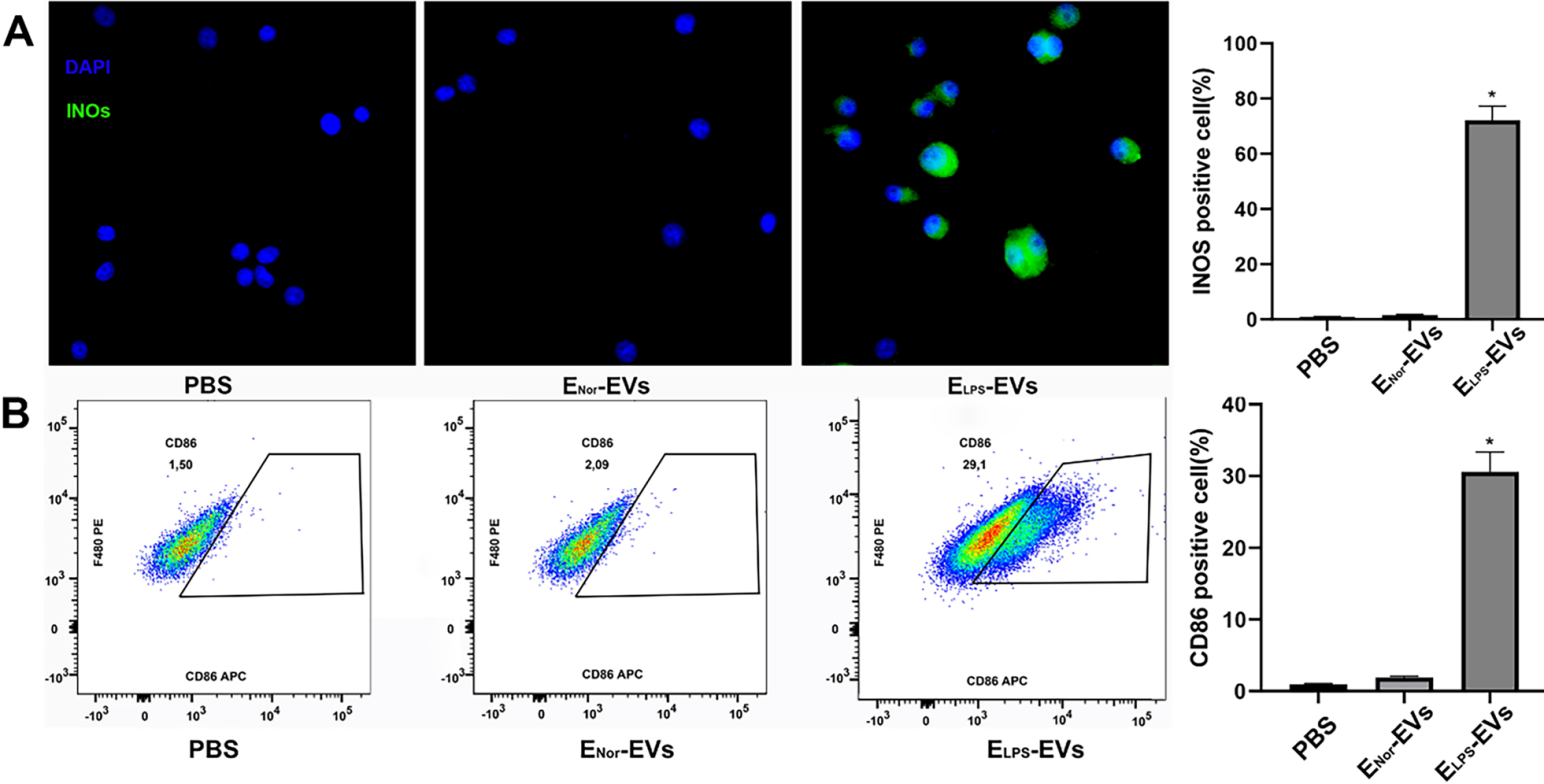
iNOS expression in and CD86 expression on NR8383 macrophages upon 24 h stimulation with E-EVs. **A** Immunofluorescence analysis of the iNOS expression in NR8383 macrophages after stimulation with either plain PBS, E_LPS_-EVs, or E_Nor_-EVs. iNOS (green fluorescence) was detected in E_LPS_-EVs treated cells, while it could not be concluded in PBS nor E_Nor_-EVs treated cells. DAPI (blue fluorescence) indicates cellular nuclei. Used magnification was × 400. n = 3 replicates were used for these experiments. *p < 0.0001 for E_LPS_-EVs compared with the PBS and E_Nor_-EVs groups. Statistical analysis was performed by one‐way ANOVA with Bonferroni's correction. Scale bar: 30 µm. **B** Flow cytometry analysis of CD86 expression on NR8383 macrophages upon stimulation with either plain PBS, E_LPS_-EVs, or E_Nor_-EVs. CD86 expression significantly increased on NR8383 macrophages as a result of the E_LPS_-EVs stimulation when compared to the expression levels of the PBS and E_Nor_-EVs groups. CD86 expression quantification is represented as average in % + SEM (n = 3 replicates). *p < 0.0001 for E_LPS_-EVs compared with the PBS and E_Nor_-EVs groups. Statistical analysis was performed by one‐way ANOVA with Bonferroni's correction

Proinflammatory markers iNOS and CD86 were analyzed by immunofluorescence analysis (Fig. 4A) and FACS (Fig. 4B), respectively. iNOS and CD86 were found in NR8383 macrophages after stimulation with E_LPS_-EVs. The expression of iNOS and CD86 was significantly higher in the E_LPS_-EVs group when compared to E_Nor_-EVs or PBS groups (*p* < 0.0001, Fig. 4). In fact, the expression of iNOS and CD86 for NR8383 macrophages stimulated with E_LPS_-EVs showed a 75 ± 5.0% and 29.1 ± 5.0% increase, respectively, as shown by immunofluorescence and FACS. This may indicate that E_LPS_-EVs stimulated macrophages polarized into M1 macrophages to a significant extend.

### ELPS-EVs activated the TLR4–NFκB signal pathway on NR8383 macrophages

**Fig. 5:**
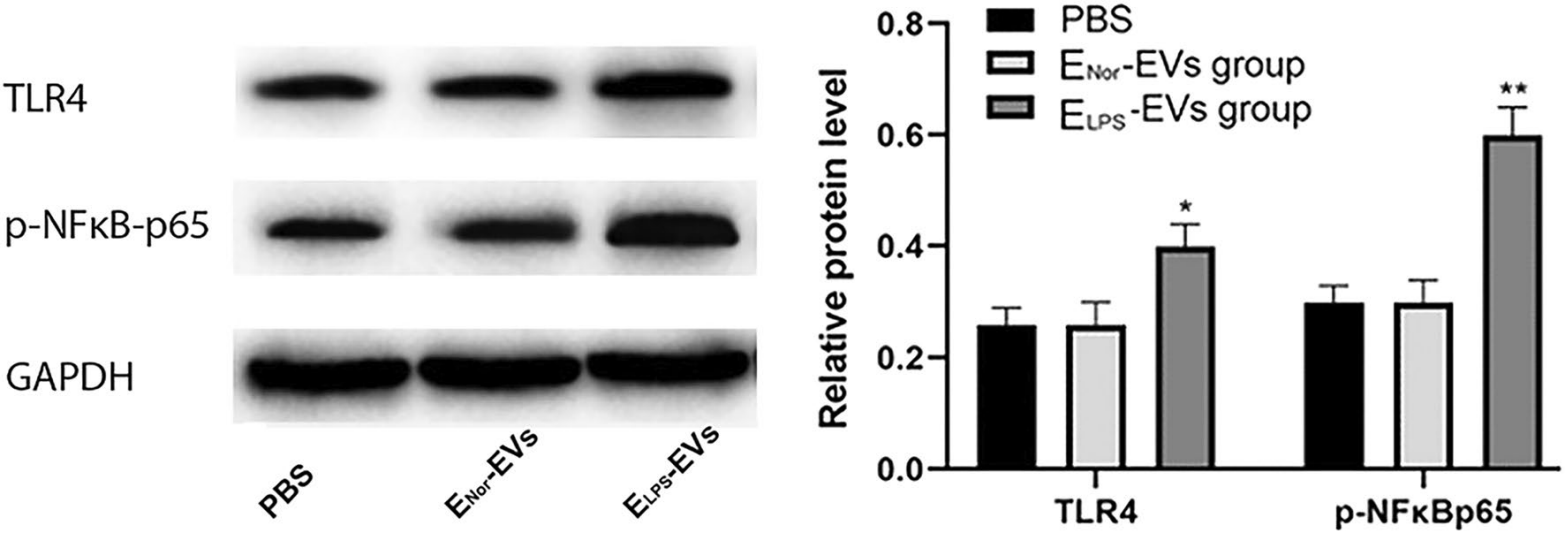
Detection of TLR4–NFκBp65 in NR8383 macrophages upon stimulation with E-EVs. Western blot analysis was used to determine the expression levels of TLR4–NFκBp65 in NR8383 macrophages treated with plain PBS, E_LPS_-EVs, or E_Nor_-EVs for 24 h. TLR4 and p-NFκBp65 expression levels were standardized by GAPDH. The protein level significantly increased (*p < 0.05 for TLR4 and **p < 0.01 for p-NFkBp65) upon stimulation of NR8383 macrophages with E_LPS_-EVs. Contrarily, no differences were concluded in the protein expression levels after E_Nor_-EVs stimulation when compared to PBS (*p* > 0.05). n = 3 replicates were used. Statistical analysis was performed by Student's *t*-test

The possible macrophage TLR4–NFκB activation by E-EVs was investigated by analyzing the protein levels on macrophages upon incubation with E_LPS_-EVs, E_Nor_-EVs, or with plain PBS. Western blot analysis showed the activation of the TLR4–NFκB pathway in macrophages exposed to E_LPS_-EVs. The proteins TLR4 and p-NFκB-p65 were significantly upregulated in NR8383 macrophages that were stimulated with E_LPS_-EVs (Fig. 5). However, E_Nor_-EVs failed to activate the TLR4–NFκB signal pathway on NR8383 macrophages. Results for the E_Nor_-EVs group were comparable to the plain PBS control group (p > 0.05 Fig. 5). The original western blot images are available in the supplementary materials (Additional file 1: Figure S1).

## Discussion

In the present study, we demonstrated that E-EVs derived from LPS-induced endothelial cells but not from quiescent endothelial cells are able to activate NR8383 macrophages to a proinflammatory status, thereby facilitating an inflammatory response. Our study additionally showed that the NFκB pathway is associated with the inflammatory phenotype changes observed on NR8383 macrophages upon stimulation with E_LPS_-EVs. This corresponds to the E-EV fraction derived from an inflammatory background investigated in this work.

The vascular endothelium is composed by the inner surface of blood vessels, a physical barrier that separates the blood from the surrounding tissues. Upon infectious microvascular injury, the function of endothelial cells significantly shifts to the recruitment of infiltrating leukocytes for immune defense [28, 29]. Details of this cell–cell communication pathway are not yet fully understood. Previous studies observed that quiescent endothelial cells suppress monocyte activation and inhibit the secretion of proinflammatory mediators [3]. The release of exosomes by monocytes and endothelial cells is known to mediate their intercellular interaction under high D-glucose conditions [30]. Despite that, to the best of our knowledge, no prior research has concentrated on the consequences of LPS-stimulated E-EVs on macrophage polarization.

Taking into account relevant features to characterize EVs, such as size, density, component, and isolation methodology [31], in this study, we separated an EV subpopulation with a size of less than 1 µm and without platelets, apoptotic bodies, and other cell detritus. The successful identification of the isolated EVs and their relevant features confirmed by TEM, NTA, and western blot, were in line with previously reported research [17–19, 32, 33] and in accordance with the guidelines published in MISEV2018 [34].

Inflammatory cytokines, endotoxin, injuries, or infectious vascular diseases are known to enhance E-EV shedding [35–37]. Cultured endothelial cells, for example, are known to shed EVs upon stimulation with LPS [17–19, 33]. A significant increase in EVs shed by these cells upon LPS stimulation was confirmed by our findings. Our results further suggested that a higher number of E_LPS_-EVs were transferred into or taken up by NR8383 macrophages when compared to E_Nor_-EVs.

E-EVs are significantly associated with inflammatory disorders and a few studies have examined the involvement of E-EVs in the fate of macrophages [3, 13, 21]. However, to date, there has not been a further evaluation of the function and underlying mechanism of E_LPS_-EVs on macrophage polarization and activation. Here we demonstrated that E_LPS_-EVs could be incorporated to a greater extent by macrophages than unstimulated E-EVs. Our EV uptake assay showed that more E_LPS_-EVs were transported into macrophages than E_Nor_-EVs within the same period of time. Possible reasons may be attributed to the heterogeneity between these two EV populations, such as the difference in cargo materials, surface protein expression, and size distribution, among others. These differences may largely determine the ability of EVs to bind and transport their cargo into recipient cells [29, 38]. These possible reasons need further detailed exploration.

LPS-stimulated endothelial cells are known to release proinflammatory EVs in high concentrations, thus promoting the proliferation of artery smooth muscle cells [17], lung endothelial cells barrier disruption [18], and VEGF-B expression in pericytes/vascular smooth muscle cells [19]. Consistent with these results, in our study, the elevated expression of proinflammatory markers CD86 and iNOS found in NR8383 macrophages upon stimulation with E_LPS_-EVs indicate a shift into the M1 status. In addition, our results also revealed that E_LPS_EVs could activate the TLR4–NFκB pathway in these cells, consistent with the previously mentioned study [3, 22–24]. The TLR4–NFκB pathway might be crucial in macrophage shifting into the proinflammatory phenotype. Along with these findings, stimulation with E_LPS_-EVs significantly favored the viability of the stimulated macrophages, indicating a positive effect.

The lack of action of E_Nor_-EVs found in macrophages might be attributed to the presence of anti-inflammatory cargo (e.g., microRNAs) in the unstimulated E-EVs that could be inhibiting the proinflammatory NFκB pathway of macrophages [3]. It might also be related to the insufficient amount of E_Nor_-EVs incorporated into the macrophages due to a short period of time. To further evaluate the role of E_Nor_-EVs on macrophages, longer stimulation time should be consider in future research.

Above all, our results suggest that the TLR4–NFκB signaling pathways might play an essential role in the changes of viability and phenotype differentiation of NR8383 macrophages induced by E_LPS_-EVs.

Although our study answered important questions regarding E-EVs uptake and impact on macrophage protein expression and morphological changes, it features important limitations. We elucidated the difference in transport of E-EVs into NR8383 macrophages when using E_LPS_-EVs compared to E_Nor_-EVs. However, the definite underlying mechanisms, such as cargo heterogeneity and membrane-bound proteins for binding, remain unknown. In addition, we investigated the TLR4–NFκB pathway as crucial in macrophage phenotype shifting. Nevertheless, other signal pathways known to regulate macrophage polarization should also be investigated in further studies in connection to LPS-derived E-EVs. Future in vivo experiments are required to elucidate the composition and mechanism of these E-EVs interacting with macrophages on infectious stimuli.

## Conclusions

In this study, we explored the intermediation of cell–cell communication between RAOECs and NR8383 macrophages via the underlying mechanism of E-EVs in an inflammatory environment. The LPS stimuli significantly upregulated endothelial cells’ EVs shedding compared to quiescent endothelial cells. Compared to the E_Nor_-EVs, E_LPS_-EVs (shed by the LPS-stimulated endothelial cells) were transferred into macrophages to a greater extent. As a result, macrophages shifted to a proinflammatory phenotype and cell viability was not affected. Although further research is needed to investigate E-EVs involvement in macrophage recruitment and polarization in infectious diseases, this study supports the notion that E-EVs represent a novel therapeutic tool to modulate immune responses and patient outcomes.

### Supplementary Information

**Additional file 1: **Original western blot images for the evaluated EV protein markers, TLR4–NFκB signal pathway protein markers, and housekeeping protein marker.

## Data Availability

All data sets are available from the corresponding author upon reasonable request.

## Abbreviations

EVs: Extracellular vesicles
E-EVs: Endothelial cell-derived EVs
E_LPS_-EVs: LPS-induced E-EVs
E_Nor_-EVs: EVs from quiescent endothelial cells
LPS: Lipopolysaccharide
TEM: Transmission electron microscopy
NTA: Nanoparticle tracking analysis
PBS: Phosphate buffered saline
iNOS: Inducible nitric oxide synthase
TLR4: Toll-like receptor 4
NFκB: Nuclear factor kappa B
p-NFκBp65: Phosphorylated form of nuclear factor kappa B p65 subunit
