# Correction: Extracellular vesicles derived from endothelial cells modulate macrophage phenotype in vitro

**DOI:** 10.1186/s40001-023-01567-9

**Published:** 2023-12-11

**Authors:** Zhizhen He, Johannes Greven, Yulong Shi, Kang Qin, Qun Zhao, Xing Zhang, Eva Miriam Buhl, Jörg Eschweiler, Frank Hildebrand, Elizabeth Rosado Balmayor

**Affiliations:** 1 Department of Orthopedics, Trauma and Reconstructive Surgery, RWTH Aachen University Hospital, Pauwelsstraße 30, 52074 Aachen, Germany; 2 Division of Joint Surgery and Sports Medicine, Department of Orthopedic Surgery, Zhongnan Hospital of Wuhan University, Wuhan, 430071 China; 3 Electron Microscopy Facility, Institute for Pathology, RWTH Aachen University Hospital, Pauwelsstraße 30, 52074 Aachen, Germany; 4 Experimental Orthopaedics and Trauma Surgery, RWTH Aachen University Hospital, Pauwelsstraße 30, 52074 Aachen, Germany

**Correction: European Journal of Medical Research (2023) 28:506** 10.1186/s40001-023-01427-6

In the original publication of the article, the Fig. 3 was duplicated as Fig. 1 inadvertently. The corrected Fig. 1 is given below. The original [1] article has been corrected.

**Fig. 1:**
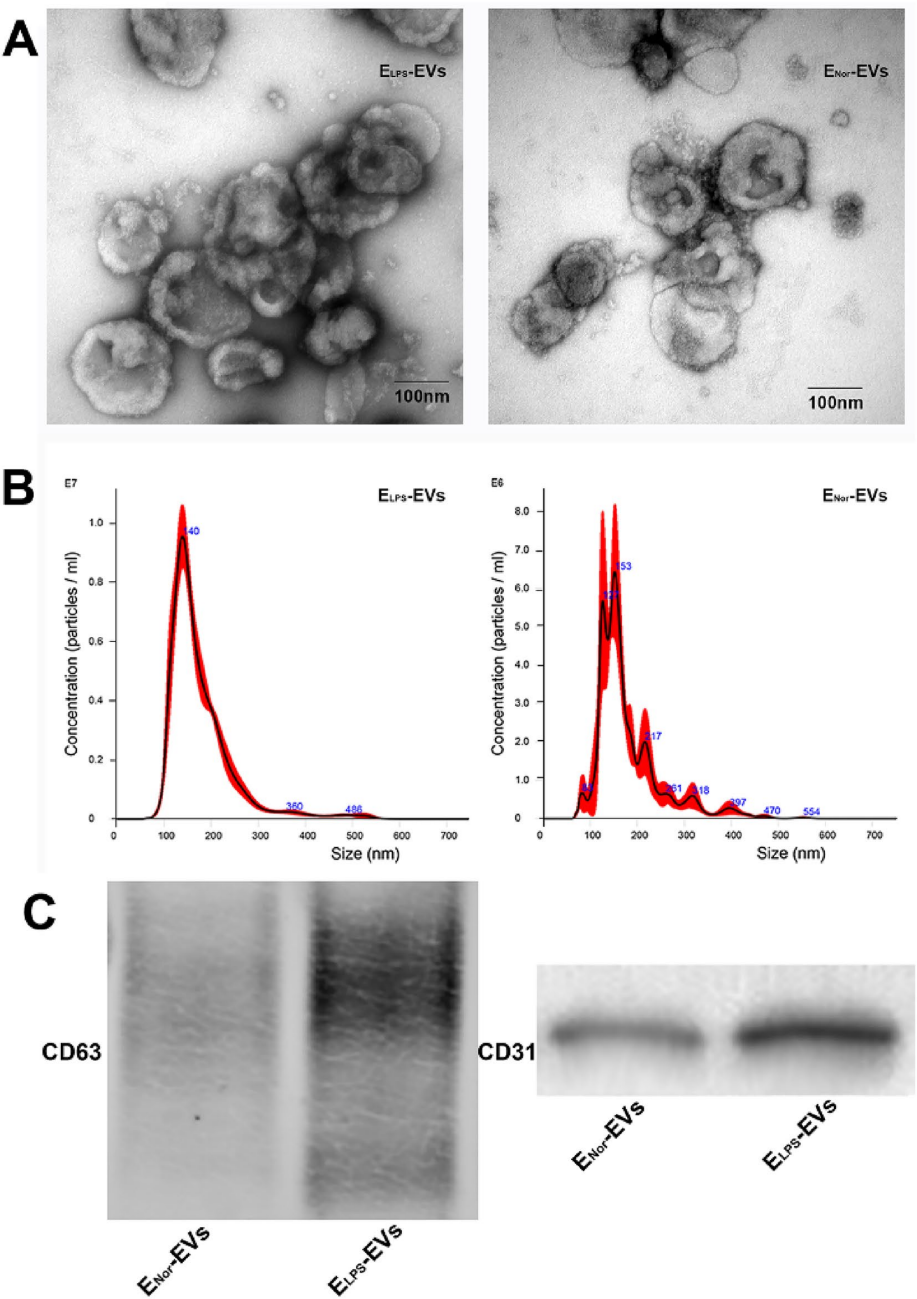
Characterization of isolated E-EVs. **A** TEM images of a representative sample of the isolated ELPS- EVs and ENor-EVs.Micrographs revealed a circular and double-membrane structure characteristic of EVs. **B** NTA determinations of size (nm)and concentration (particles/ml) of isolated ELPS-EVs and ENor-EVs. **C** Identification of EVs protein markers CD63 and CD31
