# Supplementary figures and images for: Extracellular vesicles derived from endothelial cells modulate macrophage phenotype in vitro
Source: Eur J Med Res. 2023 Nov 9;28:506. doi: 10.1186/s40001-023-01427-6 (PMC10634087; doi:10.1186/s40001-023-01427-6)

GAPDH

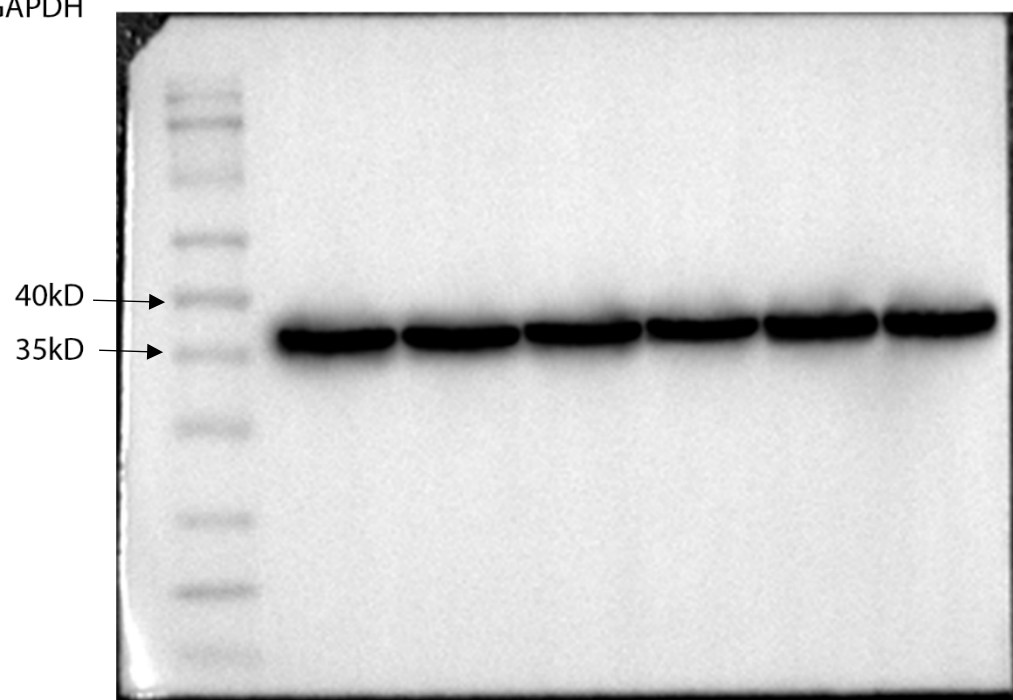

TLR4

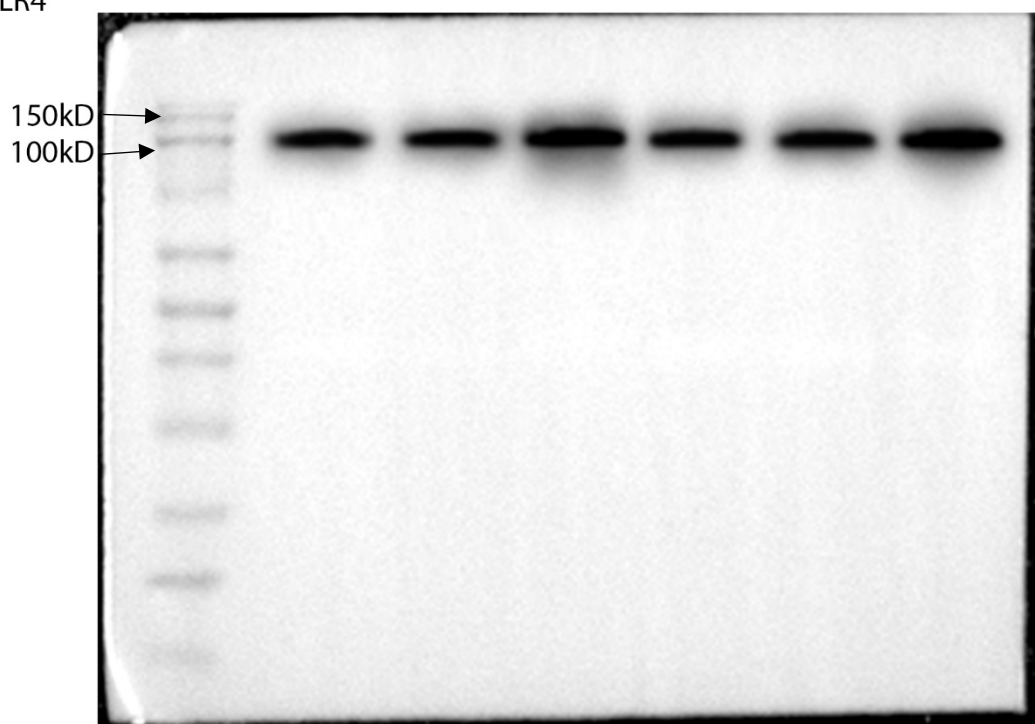

p-NFκB-p65

70kD →

50kD →

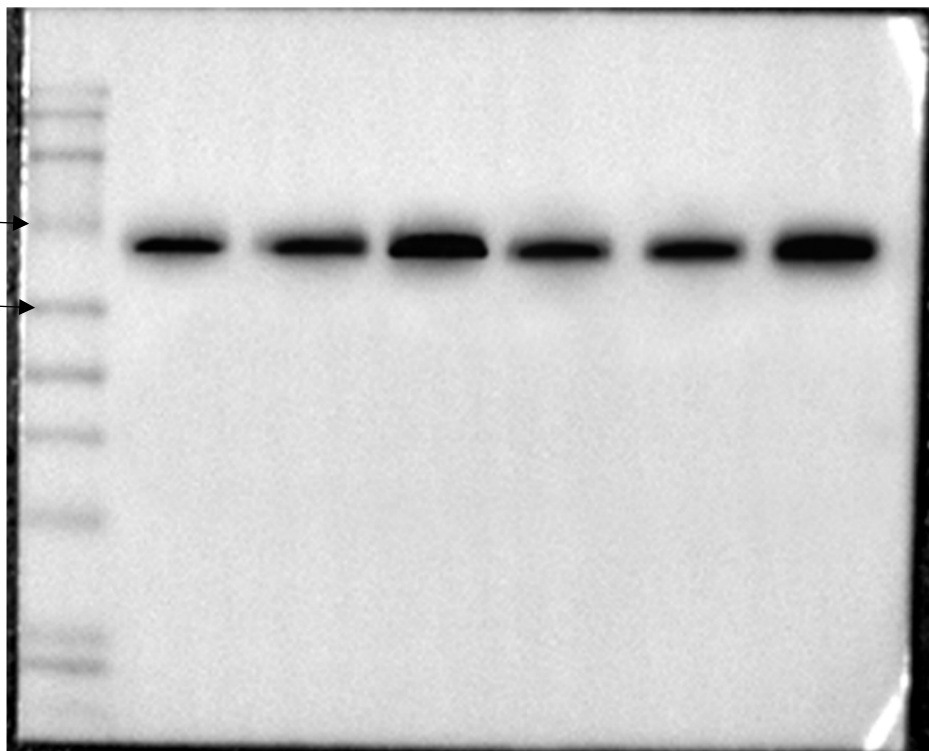

CD31

150kD →  
100kD →  
70kD →

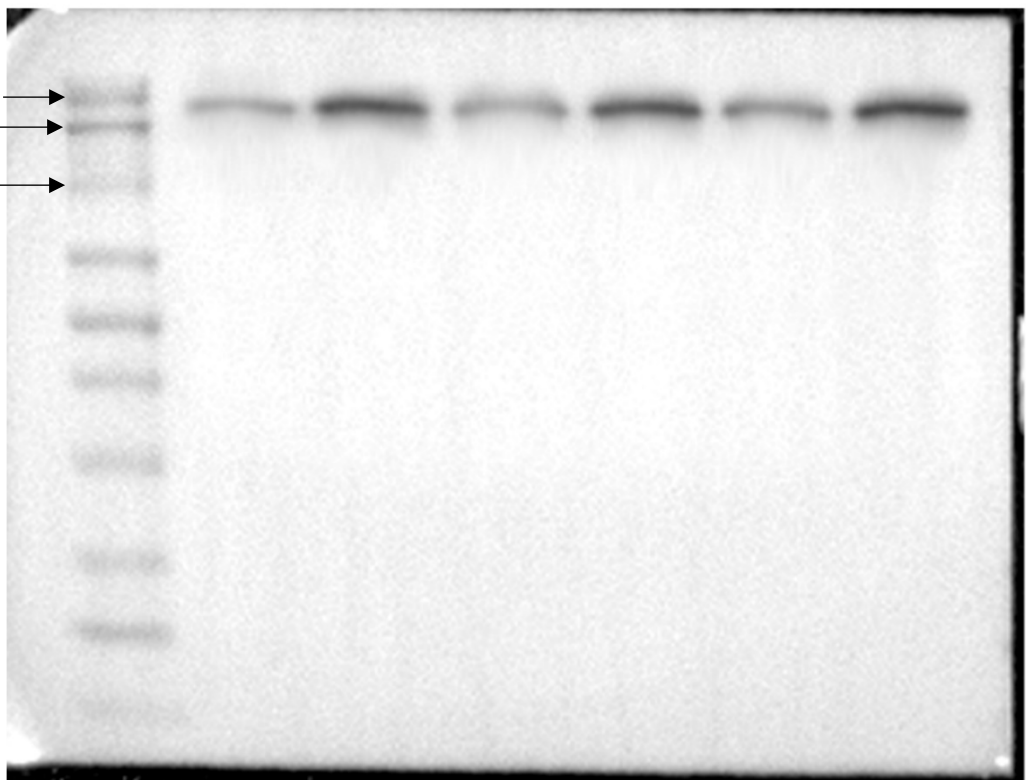

CD63

60kD →  
40kD →

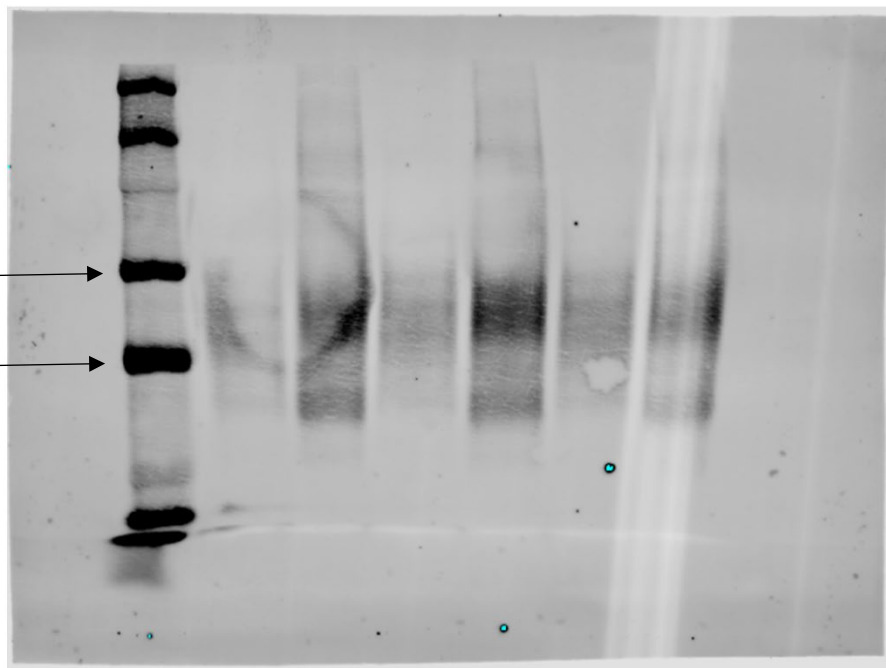

Supplement: Supplementary file 1 — Additional file 1: Original western blot images for the evaluated EV protein markers, TLR4–NFκB signal pathway protein markers, and housekeeping protein marker. [file 40001_2023_1427_MOESM1_ESM.pdf]
